# Suppression of PTBP1 in hippocampal astrocytes promotes neurogenesis and ameliorates recognition memory in mice with cerebral ischemia

**DOI:** 10.1038/s41598-024-71212-w

**Published:** 2024-09-03

**Authors:** Yusuke Fukui, Ryuta Morihara, Xinran Hu, Yumiko Nakano, Taijun Yunoki, Mami Takemoto, Koji Abe, Toru Yamashita

**Affiliations:** Department of Neurology, Graduate School of Medicine, Dentistry and Pharmaceutical Sciences, Okayama University, 2-5-1 Shikatacho, Kitaku, Okayama 700-8558 Japan

**Keywords:** CasRx, Hippocampal neurogenesis, In vivo direct reprogramming, Ischemic stroke, PHP.eB, Ptbp1, Recognition memory, Biological techniques, Neuroscience, Molecular medicine, Neurology

## Abstract

The therapeutic potential of suppressing polypyrimidine tract-binding protein 1 (Ptbp1) messenger RNA by viral transduction in a post-stroke dementia mouse model has not yet been examined. In this study, 3 days after cerebral ischemia, we injected a viral vector cocktail containing adeno-associated virus (AAV)-pGFAP-mCherry and AAV-pGFAP-CasRx (control vector) or a cocktail of AAV-pGFAP-mCherry and AAV-pGFAP-CasRx-SgRNA-(Ptbp1) (1:5, 1.0 × 10^11^ viral genomes) into post-stroke mice via the tail vein. We observed new mCherry/NeuN double-positive neuron-like cells in the hippocampus 56 days after cerebral ischemia. A portion of mCherry/GFAP double-positive astrocyte-like glia could have been converted into new mCherry/NeuN double-positive neuron-like cells with morphological changes. The new neuronal cells integrated into the dentate gyrus and recognition memory was significantly ameliorated. These results demonstrated that the in vivo conversion of hippocampal astrocyte-like glia into functional new neurons by the suppression of Ptbp1 might be a therapeutic strategy for post-stroke dementia.

## Introduction

The rates of mortality and disability from stroke have increased dramatically because of the increased numbers and age of the global population over the past several decades^1^. Acute cognitive dysfunction after stroke is well known, and the recovery of cognitive function is slower than the recovery of motor symptoms^2,3^. Stroke is estimated to hasten the onset of dementia by approximately 10 years^4^. Long-term follow-up studies of 6–12 years have reported that persistent and more rapid cognitive decline, even when corrected for its pre-stroke cognitive decline rate, also resulted in increased mortality rates^5^. Post-stroke dementia can be divided into two categories: one caused by damage to areas directly involved in cognitive function related to stroke, and the other by neuronal death in the hippocampus caused by reactive oxygen species (ROS) and other factors generated by the resumption of blood flow associated with treatments^6,7^. It has been reported that while ischemic events can cause neurogenesis in the subgranular zone of the hippocampus and the subventricular zone of the lateral ventricles transiently, the majority of newborn neurons die within 2 weeks after stroke and only 2% of mature neurons are replaced poststroke^8,9^. Although the antioxidant edaravone is already used in clinical practice to alleviate the neuronal death by ROS, effective treatment for post-stroke dementia remains to be established.

During the first few weeks after a stroke, neuroplasticity is enhanced by changes in gene and protein expression. Therefore, in addition to rehabilitation training, the upregulation of intrinsic neurogenesis is considered a very important phenomenon in stroke treatment^10,11^. The RNA-binding protein polypyrimidine tract-binding protein 1 (Ptbp1) was reported to repress neuron-specific splicing and be expressed at high levels in non-neuronal cells but downregulated in neurons^12^. The suppression of Ptbp1 expression derepressed many neuronal genes in non-neuronal cells and induced cellular reprogramming of cultured mouse fibroblasts and N2a cells toward the neuronal lineage^13^. A recent study reported that the transient suppression of Ptbp1 using an antisense oligonucleotide administered intracerebroventricularly (i.c.v.) successfully activated hippocampal neurogenesis and generated new neurons in the dentate gyrus (DG) of adult mice^14^. However, i.c.v. injection is highly invasive, and its effects on the diseased brain remain unknown because it is normally used for healthy young and aged wild-type mice. In the present study, therefore, we examined the therapeutic potential of suppressing Ptbp1 messenger RNA (mRNA) by viral transduction in a post-stroke dementia mouse model^15^.

## Results

To confirm the distribution of the viral vectors, we injected AAV(PHP.eB)-pGFAP-mCherry into mice via the tail vein (Fig. S1a). mCherry positive cells were detectable throughout the whole brain and were mainly present in cells positive for GFAP, an astrocytic marker (57.5% = 163 cells/283 mCherry positive cells from nine brain sections of the cortex from three mice) and S100β (astrocytic marker; 57.0% = 106 cells/186 mCherry positive cells). Fewer mCherry positive cells were detected in cells positively stained for Tuj1 (neuronal marker; 8.5% = 17 cells/200 mCherry positive cells), MAP2 (13.7% = 32 cells/232 mCherry positive cells), NeuN (13.9% = 48 cells/345 mCherry positive cells), and Iba1 (a microglial marker; 0.4% = 1 cells/274 mCherry positive cells). However, the mCherry positive cells did not coexpress the oligodendrocyte marker Olig2 (0% = 0 cells/201 mCherry positive cells) (Fig. S1b). A quantitative analysis of the number of positive cells for mCherry and each marker was conducted. No significant differences were observed in the hippocampus (GFAP: 61.7 ± 14.7 cells/0.54 mm^2^, S100β: 18.3 ± 5.2 cells/0.54 mm^2^, Iba1: 30.7 ± 4.9 cells/0.54 mm^2^, Tuj1: 60.7 ± 14.1 cells/0.54 mm^2^, MAP2: 68.0 ± 12.8 cells/0.54 mm^2^, NeuN: 67.0 ± 15.3 cells/0.54 mm^2^). However, significant differences were identified between GFAP and Iba1 (*p < 0.05), as well as between GFAP and Olig2 (*p < 0.05), in the cortex (GFAP: 54.3 ± 15.7 cells/0.54 mm^2^, S100β: 35.3 ± 6.9 cells/0.54 mm^2^, Iba1: 0.3 ± 0.5 cells/0.54 mm^2^, Tuj1: 5.7 ± 1.9 cells/0.54 mm^2^, MAP2: 10.7 ± 3.1 cells/0.54 mm^2^, NeuN: 12.3 ± 3.4 cells/0.54 mm^2^) (Fig. S1b).

Next, we injected a cocktail of AAV(PHP.eB)-pGFAP-mCherry and AAV(PHP.eB)-pGFAP-CasRx-Ptbp1 at 3, 7, or 14 days after 30 min tMCAO to evaluate the appropriate time point for viral injection (Fig. S2a). The pixel intensity of mCherry showed a significant time-dependent decrease on day 7 (0.74 ± 0.06 pixels fold change to day 3) or day 14 (0.31 ± 0.09 pixels fold change to day 3) (Fig. S2b). On the basis of these results, day 3 was selected as the appropriate time point for viral injection in the following experiments.

**Fig. 1:**
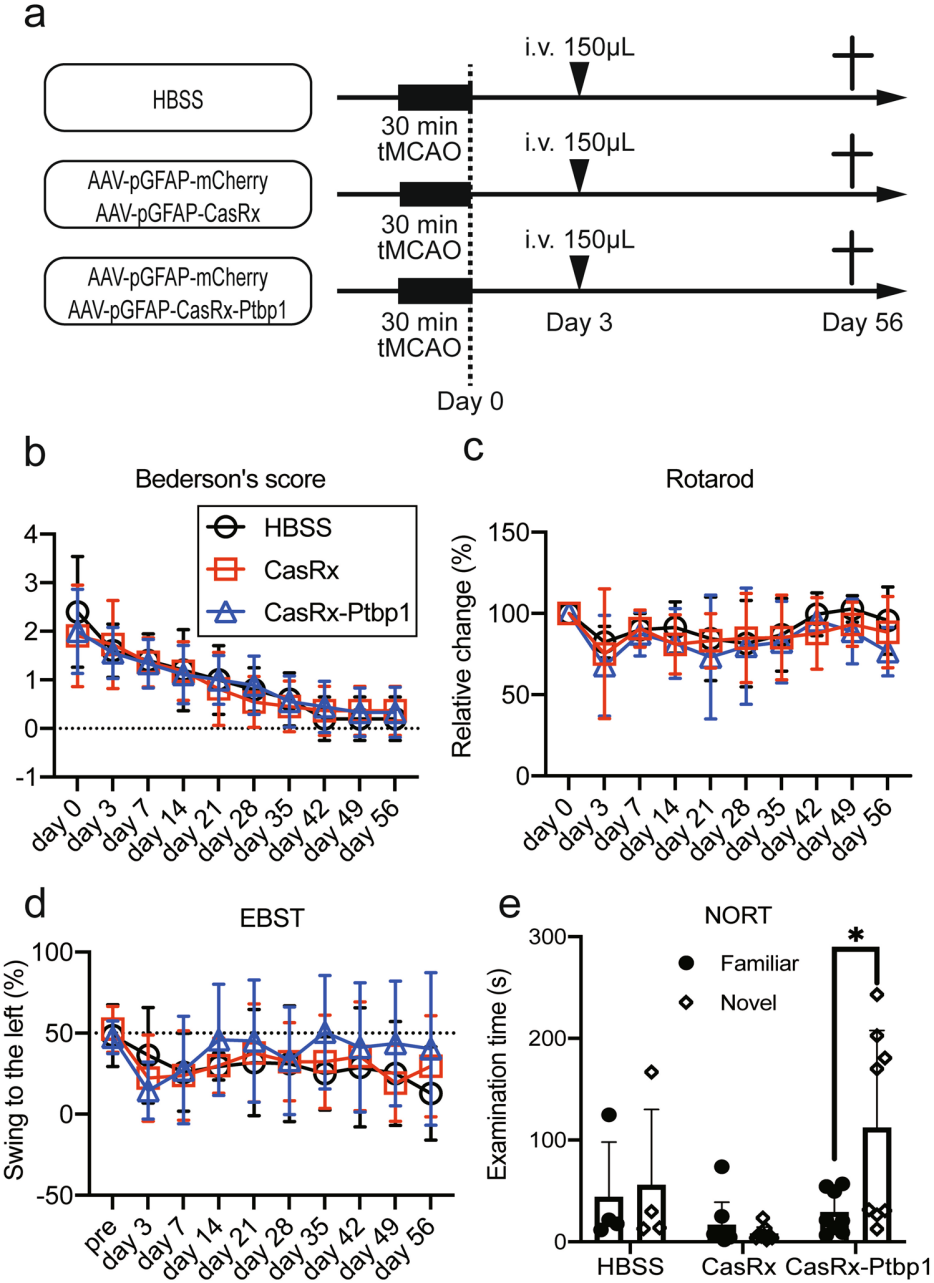
(**a**) Schematic diagram of the experimental procedure. The arrowheads indicate the intravenous injection of HBSS, a cocktail of AAV(PHP.eB)-pGFAP-mCherry and AAV(PHP.eB)-pGFAP-CasRx as a control vector, or a cocktail of AAV(PHP.eB)-pGFAP-mCherry and AAV(PHP.eB)-pGFAP-CasRx-SgRNA-Ptbp1. (**b**) Bederson’s score, (**c**) the rotarod test, and (**d**) elevated body swing test (EBST) were compared between the three groups. (**e**) The examination time between a novel object and a familiar object in the novel object recognition test (NORT) was compared between groups. Note that the CasRx-Ptbp1 group showed a significant increase in the novel object time compared with the familiar object time (*p < 0.05). *P*-values < 0.05 were considered statistically significant.

**Fig. 2:**
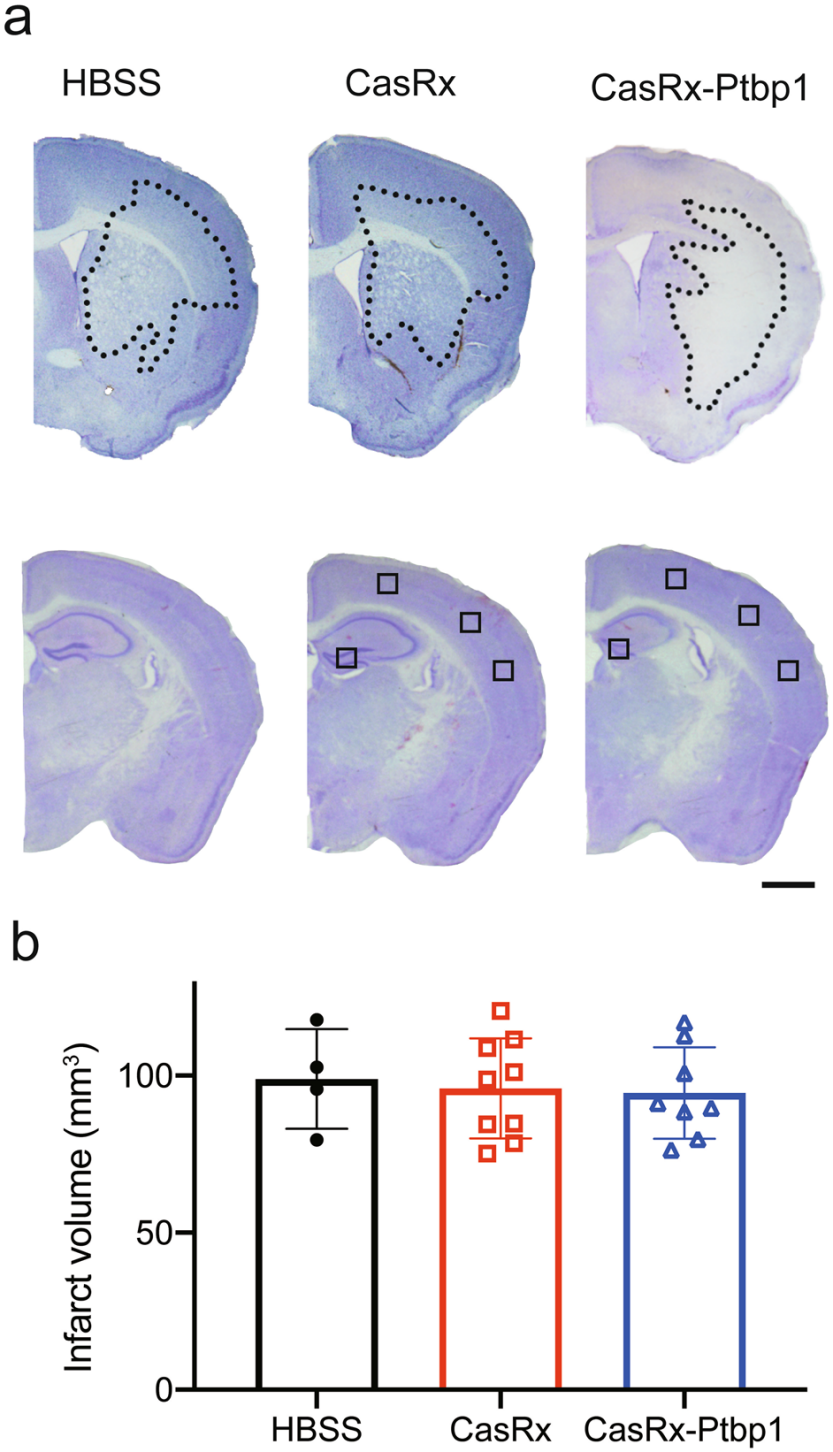
(**a**) Nissl staining and infarct volume 56 days after tMCAO in the three experimental groups. The use of small squares indicates the approximate area where imaging was performed. Scale bars: 1 mm. (**b**) Quantitative analysis of infarct volume in in the three experimental groups.

**Fig. 3:**
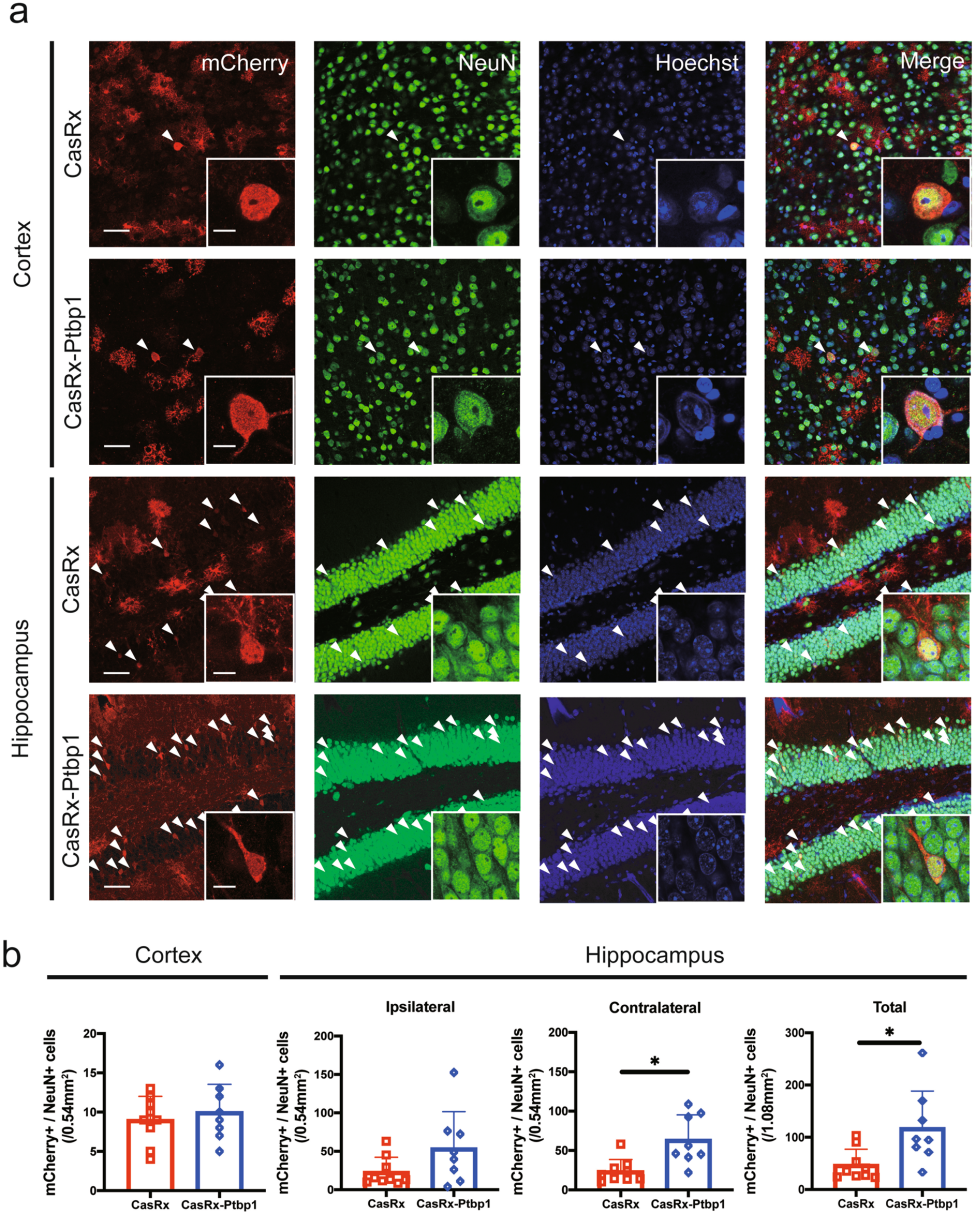
(**a**) Immunofluorescent staining of mCherry/NeuN/Hoechst in the cortex and hippocampus 56 days after tMCAO. Arrowheads indicate mCherry/NeuN double-positive cells. Scale bars: 50 µm and 10 µm. (**b**) Quantitative analysis of mCherry and NeuN double-positive cells in the cortex and hippocampus. CasRx-Ptbp1 significantly increased the number of mCherry/NeuN double-positive cells in the DG compared with the CasRx group (*p < 0.05). *P*-values < 0.05 were considered statistically significant.

**Fig. 4:**
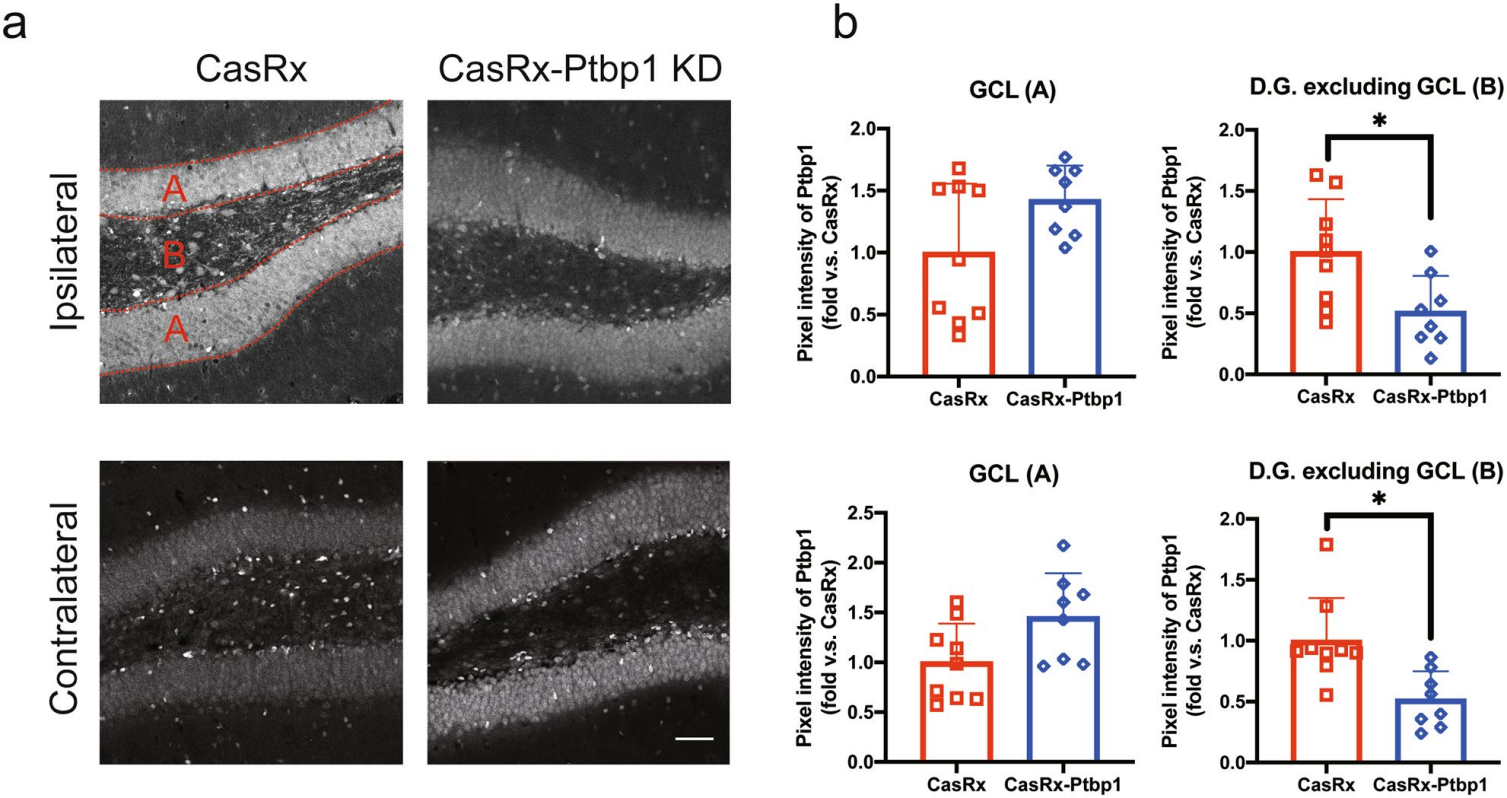
(**a**) Representative images of Ptbp1 (white) in the hippocampus of the two experimental groups. Scale bars: 50 µm. (**b**) Pixel intensity of Ptbp1 in GCL indicated by A and the DG excluding the GCL indicated by B. The Ptbp1 expression was significantly lower in CasRx-Ptbp1 in DG excluding GCL. (*p < 0.05) *P*-values < 0.05 were considered statistically significant.

Next, we injected HBSS, a cocktail of AAV(PHP.eB)-pGFAP-mCherry and AAV(PHP.eB)-pGFAP-CasRx as a control vector, or a cocktail of AAV(PHP.eB)-pGFAP-mCherry and AAV(PHP.eB)-pGFAP-CasRx-SgRNA-Ptbp1 via the tail vein 3 days after 30 min tMCAO (Fig. 1a). We checked Bederson’s score, the rotarod test, and EBST to confirm improvements in motor function; however, there were no significant differences between the three groups (Fig. 1b–e). The NORT, which is associated with hippocampal recognition memory, was performed 56 days after tMCAO. There was no significant difference in the time to approach a familiar object between the three groups (HBSS: 43.9 ± 54.1 s, CasRx: 16.4 ± 22.4 s, CasRx-Ptbp1: 29.1 ± 21.1 s). Of note, only the CasRx-Ptbp1 group showed a significant increase in the novel object time compared with the familiar object time (HBSS: 55.8 ± 74.4 s, CasRx: 8.0 ± 6.6 s, CasRx-Ptbp1: 112.2 ± 95.5 s, *p < 0.05) (Fig. 1e). Next, we checked the cerebral ischemic volume, but there was no significant difference in ischemic volume between the three groups 56 days after tMCAO (HBSS: 98.9 ± 15.9 mm^3^, CasRx: 96.0 ± 15.9 mm^3^, CasRx-Ptbp1: 94.5 ± 14.8 mm^3^) (Fig. 2a,b). We also checked for alterations in hippocampal neurons (CA1, CA2, and CA3), but there were no significant differences in the ischemic side and non-ischemic side between the three groups (Figs. S3, S4). In addition, there was no significant increase in the number of mCherry/NeuN double-positive cells in the cortex between the CasRx and CasRx-Ptbp1 groups (CasRx: 9.0 ± 3.0 cells/0.54 mm^2^, CasRx-Ptbp1: 10.0 ± 3.5 cells/0.54 mm^2^). However, there were significantly more mCherry/NeuN double-positive cells in the DG of the CasRx-Ptbp1 group compared with the CasRx group (Total; CasRx: 47.1 ± 30.1 cells/1.08 mm^2^, CasRx-Ptbp1: 117.6 ± 70.9 cells/1.08 mm^2^, *p < 0.05, Ipsilateral; CasRx: 23.2 ± 17.9 cells/0.54 mm^2^, CasRx-Ptbp1: 53.9 ± 44.6 cells/0.54 mm^2^, Contralatral; CasRx: 23.8 ± 14.0 cells/0.54 mm^2^, CasRx-Ptbp1: 63.7 ± 29.5 cells/0.54 mm^2^, *p < 0.05) (Fig. 3a, b). We next confirmed Ptbp1 expression in the hippocampal granule cell layer (GCL) indicated by A in Fig. 4a and in the DG excluding the GCL indicated by B in Fig. 4a. The Ptbp1 is also expressed in the GCL, and no significant difference was observed between CasRx and CasRx-ptbp1 in GCL (Ipsilateral: 1.42 ± 0.26 fold change to CasRx, Contralateral: 1.46 ± 0.41 fold change to CasRx). However, in DG excluding the GCL, the Ptbp1 expression was significantly lower in CasRx-Ptbp1 (Ipsilateral: 0.60 ± 0.27 fold change to CasRx, *p < 0.05, Contralateral: 0.63 ± 0.22 fold change to CasRx, *p < 0.05) (Fig. 4a,b).

## Discussion

In the present study, we demonstrated that AAV-pGFAP-CasRx-Ptbp1 crossed the BBB and generated new mCherry/NeuN double-positive neuron-like cells, its origin could be mCherry/GFAP double-positive astrocyte-like glia, in the hippocampus of post-stroke dementia mouse model (Figs. 3a and b, S1, S2). Moreover, the CasRx-Ptbp1 group showed a significant amelioration in recognition memory on day 56 after tMCAO (Fig. 1e).

During adult hippocampal neurogenesis, neural stem cells are activated and differentiate into proliferating intermediate neural progenitors and neuroblasts, which become post-mitotic immature dentate granule cells that eventually mature and become incorporated into the neural circuitry^16^. In ischemic stroke, adult neurogenesis of the DG is markedly increased in the ipsilateral and contralateral hemispheres^11,17^. However, the division of progenitor cells starts to decrease in the second week and returns to the control level within a few weeks^18,19^. Additionally, it has been demonstrated that the number of hippocampal neurogenesis and neural progenitor cells declines with age^20–22^. Nevertheless, even in aged mice, the reduction of Ptbp1 has been observed to result in the conversion of radial glia-like cells to granule cells, a phenomenon that has been documented in young mice and during development^14^. In this study, we elucidated that hippocampal neurogenesis was robustly stimulated and significantly recovered hippocampal recognition memory in the CasRx-Ptbp1 group 56 days after tMCAO (Figs. 1e, 3a and b). miR-124, a neuron-specific microRNA, suppresses Ptbp1 protein expression by inhibiting the 3′ UTR of Ptbp1 mRNA. This suppression allows the shift to neuron-specific splicing^12^, which might promote neuronal differentiation from astrocytes, as well as change the fate of radial glia that normally convert to astrocytes, forcing neuronal differentiation^14^.

The in vivo conversion of resident glial cells to functional new neurons by the ectopic expression of a single or combination of transcription factors or by the knockdown of a single gene has been attempted by many researchers^14,23–33^. However, some of these results have been questioned by fate-mapping experiments, which are the gold standard for definitively confirming the origin of newly generated cells^34–36^. The primary cause is regarded as the leaky expression of the construct into neurons under a GFAP promoter^37^. In the present study, we observed a phenomenon considered leakage expression and could not detect a significant increase in ectopic new neurons in the cortex (Figs. 3a and b, S1). However, we could explain why hippocampal neurogenesis was increased, and hippocampal recognition memory was recovered in the CasRx-Ptbp1 group only (Figs. 1e, 3a and b). This suggests that CRISPR-CasRx-mediated Ptbp1 knockdown may impact hippocampal astrocytes and radial glia, dramatically enhancing endogenous neurogenesis.

Currently, recombinant AAVs (rAAV) have become a significant platform for the in vivo delivery of gene therapy. A combinatorial design that coordinates the genomic characteristics and properties of the capsid and the vector is needed to create an rAAV platform for treating human diseases^38^. The efficacy and safety of voretigene neparvovec, which is a new drug for RPE65-mediated inherited retinal dystrophy using AAV2 discovered in human cells, and onasemnogene abeparvovec-xioi, which is a prescription for spinal muscular atrophy packaged by AAV9, have been confirmed and used for treatment^39,40^. However, there is still room for improvement because issues remain with these gene delivery platforms, including immunological barriers to the delivery of rAAV. In the near future, this platform might be a target for treating cerebral infarction after the further improvement of the Rep/Cap packaging plasmid pAAV-PHP.eB with a GFAP promoter.

The present study has some limitations: as in previous studies^14^, we observed that hippocampal recognition memory was recovered in the CasRx-Ptbp1 group only (Figs. 1e, 3a and b). However, only one type of cognitive function test was performed, so it would be beneficial to validate this result in other cognitive tests in a future study. Secondly, we have not conducted any experiments to assess the loss of function. It would have been preferable to perform such experiments using AAV vectors carrying Designer Receptors Exclusively Activated by Designer Drugs (DREADDs) which manipulate a series of human muscarinic receptors that respond only to the synthetic ligand clozapine N-oxide (CNO)^41^. Thirdly, it has been reported that the knockdown of Ptbp1 increases the number of DCX-positive cells in the granule cell layer^14^. However, we have not directly examined whether there is an increase or decrease in neural progenitor cells. In our next study, we intend to investigate the mechanism of neurogenesis using BrdU and markers of each neural progenitor cell. Fourthly, in light of the ongoing discussions surrounding ectopic direct reprogramming, it is crucial to trace the lineage back to the origin of the cell. Our present study does not provide a definitive answer as to the precise origin of the new neurons.

In conclusion, our findings indicate that the transient suppression of Ptbp1 mRNA by viral transduction following the administration of AAV via the tail vein has the potential to facilitate the generation of new neurons from astrocyte-like glia in the hippocampus of a post-stroke dementia mouse model by the CasRx-Ptbp1 vector.

## Materials and methods

### Animals and pretreatment

Adult male C57BL/6 J mice (20–25 g, 7 weeks old) were purchased from Japan SLC Inc. (Shizuoka Japan). In the first pilot study, mice were injected with AAV(PHP.eB)-pGFAP-mCherry (4.6 × 10^10^ viral genomes (vg)) through the tail vein to evaluate whether the viral vector could cross the blood–brain barrier (BBB) and infect astrocytes in the brain. Mice were sacrificed 21 days after injection (Fig. S1). In the second pilot study, mice were injected with a cocktail of AAV(PHP.eB)-pGFAP-mCherry and AAV(PHP.eB)-pGFAP-CasRx-SgRNA-Ptbp1 (1:5, total 1.2 × 10^10^ vg) through the tail vein at 3, 7, or 14 days after 30 min transient middle cerebral artery occlusion (tMCAO) to determine the optimal time point of the viral vector injection to the post-stroke brain. Mice were sacrificed 21 days after injection (Fig. S2). In the first full experiment, mice were randomly divided into three groups depending on the treatment: HBSS group (150 µL, n = 4); cocktail of AAV(PHP.eB)-pGFAP-mCherry and AAV(PHP.eB)-pGFAP-CasRx (control vector) group (1:5, total 1.0 × 10^11^ vg, n = 9); and cocktail of AAV(PHP.eB)-pGFAP-mCherry and AAV(PHP.eB)-pGFAP-CasRx-SgRNA-Ptbp1 group (1:5, total 1.0 × 10^11^ vg, n = 8). Mice were injected through the tail vein at 3 days after 30 min tMCAO and sacrificed 56 days after the injection (Fig. 1a). Animal procedures were approved by the Animal Care and Committee of the Okayama University Graduate School of Medicine, Dentistry and Pharmaceutical Sciences (approval #OKU-2021732), and all animal experiments were conducted and reported in accordance with the Okayama University guidelines on the Care and Use of the Laboratory Animals and ARRIVE guidelines.

### Focal cerebral ischemia

Focal cerebral ischemia was induced in mice by tMCAO following our previous reports^42,43^. Briefly, mice were anesthetized with isoflurane through an inhalation mask. The left common carotid artery was exposed and a 7–0 nylon thread with a silicone-coated tip was inserted into the left middle cerebral artery (MCA). Following a 30 min period of occlusion, the silicone-coated thread was extracted in order to restore blood flow. Then, the incision was closed, and the animals recovered and were allowed free access to water and food at ambient temperature. Four sham control mice were prepared with a sham cervical operation but without inserting the thread.

### AAV vectors and virus production

AAV-GFAP-NLS-CasRx-NLS-FLAG and AAV-GFAP-NLS-CasRx-NLS-FLAG-U6-DR-SgRNA_Ptbp1-DR plasmids were obtained from Addgene (#154000 and #154001, MA, USA). Each plasmid was packed by a pHelper plasmid (#240071, Agilent Technologies, CA, USA) and the Rep/Cap packaging plasmid pAAV-PHP.eB, provided by Dr. Sehara at Jichi Medical University, in HEK293TF cells. All plasmids were verified by restriction enzyme digestion before use. Packaging plasmids and a vector plasmid were transfected into HEK293TF cells using Fugene HD (E2311, Promega, WI, USA). Culture medium containing the transfection reagent was changed to serum-free medium 1 day after transfection. The AAV-containing supernatants were collected 6 days after transfection, and AAV were purified using the minimal purification method^44^. Viral titers were confirmed using an AAVpro® Titration Kit for Real-Time PCR Ver. 2 (#6233, Takara Bio, Shiga, Japan) with primers recognizing the inverted terminal repeat (ITR) regions.

### Behavioral analysis after tMCAO

Mice were evaluated for changes in body weight and behavior at 3, 7, 14, 21, 28, 35, 42, 49, and 56 days after tMCAO. Bederson’s scale scores with minor modifications were applied in this study^23,45^. A rotarod test was also conducted to detect the impairment of motor function, which correlates with ischemic volume and neurological scores as described previously^23^. The elevated body swing test (EBST) was performed as described previously^46,47^. Briefly, the percentage of the number of left side swings, in which the mouse moved its head more than 90° from the vertical axis to either side to the total number of swings, was calculated in 60 s. The novel object recognition test (NORT) was performed to test recognition memory, which is thought to be linked to hippocampal damage^14^. The mice were habituated in a 35 × 35 × 16 cm^3^ open field for 2 h. After habituation, the mice were placed in the box with the same two objects (yellow ball with a diameter of 7 cm) for 10 min as training. After 1 h, the time taken for the mouse to approach the novel object (shuttlecock of badminton) and the familiar object was recorded, and then behavior was scored for the object contact time (Fig. 1b).

### Tissue preparation

Each mouse was anesthetized by the intraperitoneal injection of pentobarbital (20 mg/kg), and then perfused with chilled PBS, followed by 4% paraformaldehyde (PFA) in 0.1 mol/L phosphate buffer. After post-fixation in the same fixative for 12 h at 4 °C, whole brains were cut into 50-μm-thick sections with a vibrating blade microtome (VT1000S; Leica, Wetzlar, Germany).

### Infarct volume and immunofluorescent staining

Coronal brain sections were stained with cresyl violet as Nissl staining to measure the infarct and hippocampal areas using image processing software (ImageJ; National Institutes of Health, MD, USA). The infarct volume of each brain was calculated by the summation of the infarct areas of five serial brain slices, at a 0.6 mm interval, between 1.0 mm anterior and 1.5 mm posterior to the bregma^48^.

Mice exhibiting neuronal loss in Nissl-stained coronal sections, including the hippocampus, were excluded from the analysis to prevent the direct effects of cerebral ischemia on the hippocampus.

The small squares in Fig. 2 indicated the approximate analyzed area. In the cortex, three locations were randomly selected without distinction between the ipsilateral and contralateral sides. The hippocampus was analyzed separately for the ipsilateral and contralateral sides.

For immunofluorescent staining, free-floating sections were blocked in 5% bovine serum albumin for 2 h. Then, they were incubated at 4 °C overnight with primary antibodies. All antibodies used in this study were from commercial sources: rabbit anti-mCherry antibody (1:200, GTX128505, GeneTex, CA, USA), mouse anti-mCherry antibody (1:200, GTX630189, GeneTex), rabbit anti-glial fibrillary acidic protein (GFAP) antibody (1:500, Z0334, Dako, Glostrup, Denmark), rabbit anti-S100β antibody (1:200, GTX129573, GeneTex, CA, USA), rabbit anti-PTBP1 antibody (1:200, 12582-1-AP, Proteintech, IL, USA), rabbit anti-ionized calcium binding adapter protein 1 (Iba1) antibody (1:500, NCNP24, Wako, Osaka, Japan), rabbit anti-Olig-2 antibody (1:100, AB9610, Merck Millipore, MA, USA), mouse anti-beta III tubulin (Tuj1) antibody (1:100, sc-58888, Santa Cruz Biotechnology, TX, USA); rabbit anti-MAP2 (1:100, sc-58888, Santa Cruz Biotechnology), mouse anti-NeuN antibody (1:200, MAB377, Merck Millipore), rabbit anti-NeuN antibody (1:1000, ab104225, Abcam, Cambridge, UK), and mouse anti-NeuroD1 antibody (1:200, ab60704, Abcam). The sections were washed in PBS and then incubated with secondary antibodies Alexa Fluor 488 or 555 (1:500; Molecular Probes, MA, USA) for 2 h at room temperature.

### Statistical analysis

All data are presented as the mean ± standard deviation. Statistical analyses were performed using GraphPad Prism version 8.4.3 (GraphPad Software LCC, CA, USA). After having checked for normality, we performed the Mann–Whitney *U*-test to compare mCherry and NeuN positive cells in the cortex and hippocampus between the CasRx and CasRx-Ptbp1 groups, and the examination time of NORT between the novel object and familiar object. We also performed a two-way repeated measure ANOVA to compare Bederson’s score, rotarod test, and EBST between the HBSS, CasRx, and CasRx-Ptbp1 groups. Other statistical analyses were performed with the Kruskal–Wallis test to compare ischemic volume, pixel intensity of mCherry and Nissl staining between the HBSS, CasRx, and CasRx-Ptbp1 groups. *P*-values < 0.05 were considered statistically significant.

## Supplementary Information

Supplementary Figures.

## Data Availability

The datasets generated or analyzed during this study are included in this paper and its Supplementary Information file or are available from the corresponding author upon reasonable request.

## Abbreviations

AAV: Adeno-associated virus
BBB: Blood–brain barrier
EBST: Elevated body swing test
GFAP: Glial fibrillary acidic protein
Iba-1: Ionized calcium binding adapter protein 1
i.c.v.: Intracerebroventricular
MCA: Middle cerebral artery
mRNA: Messenger RNA
NORT: Novel object recognition test
PBS: Phosphate-buffered saline
PFA: Paraformaldehyde
Ptbp1: Polypyrimidine tract-binding protein 1
rAAV: Recombinant AAVs
tMCAO: Transient middle cerebral artery occlusion
Tuj1: Beta III tubulin
vg: Viral genome
