# Supplementary material for: Suppression of PTBP1 in hippocampal astrocytes promotes neurogenesis and ameliorates recognition memory in mice with cerebral ischemia
Source: Sci Rep. 2024 Sep 3;14:20521. doi: 10.1038/s41598-024-71212-w (PMC11372044; doi:10.1038/s41598-024-71212-w)
Supplement: Supplementary file 1 — Supplementary Figures. [file 41598_2024_71212_MOESM1_ESM.pdf]

Fig. S1

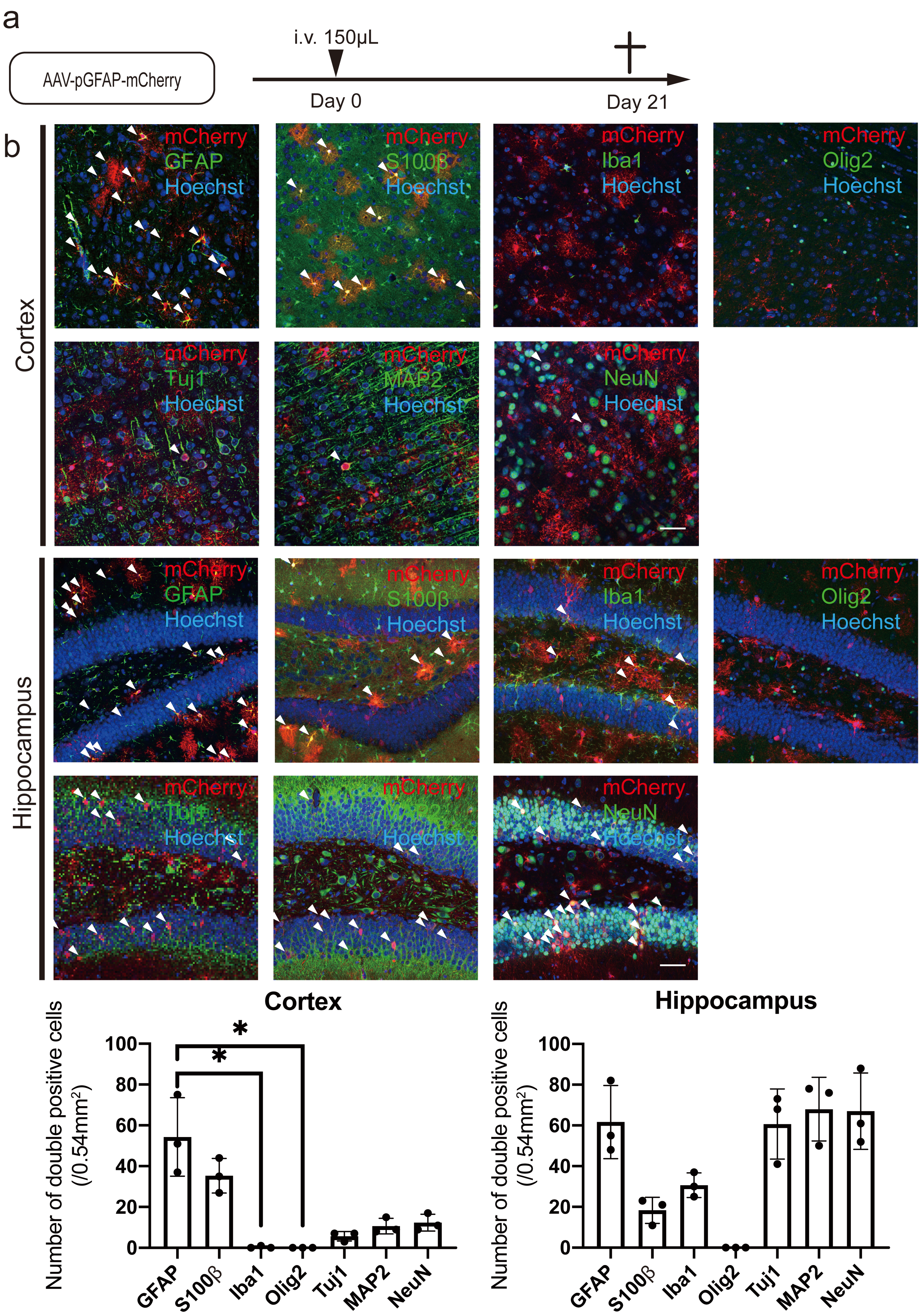

Fig. S1 (a) Schematic diagram of the experimental procedure to confirm the distribution of the viral vectors. The arrowheads indicate the intravenous injection of AAV(PHP.eB)-pGFAP-mCherry (4.6 × 10^{10} vg) through the tail vein. (b) Immunofluorescent analysis of mCherry/GFAP/Hoechst, mCherry/S100 β/Hoechst,mCherry/Iba1/Hoechst, mCherry/Olig2/Hoechst, mCherry/Tuj1/Hoechst, mCherry/Map2/Hoechst, and mCherry/NeuN/Hoechst in the cortex and hippocampus at 21 days after tMCAO. Scale bars: 50 μm.

Fig. S2

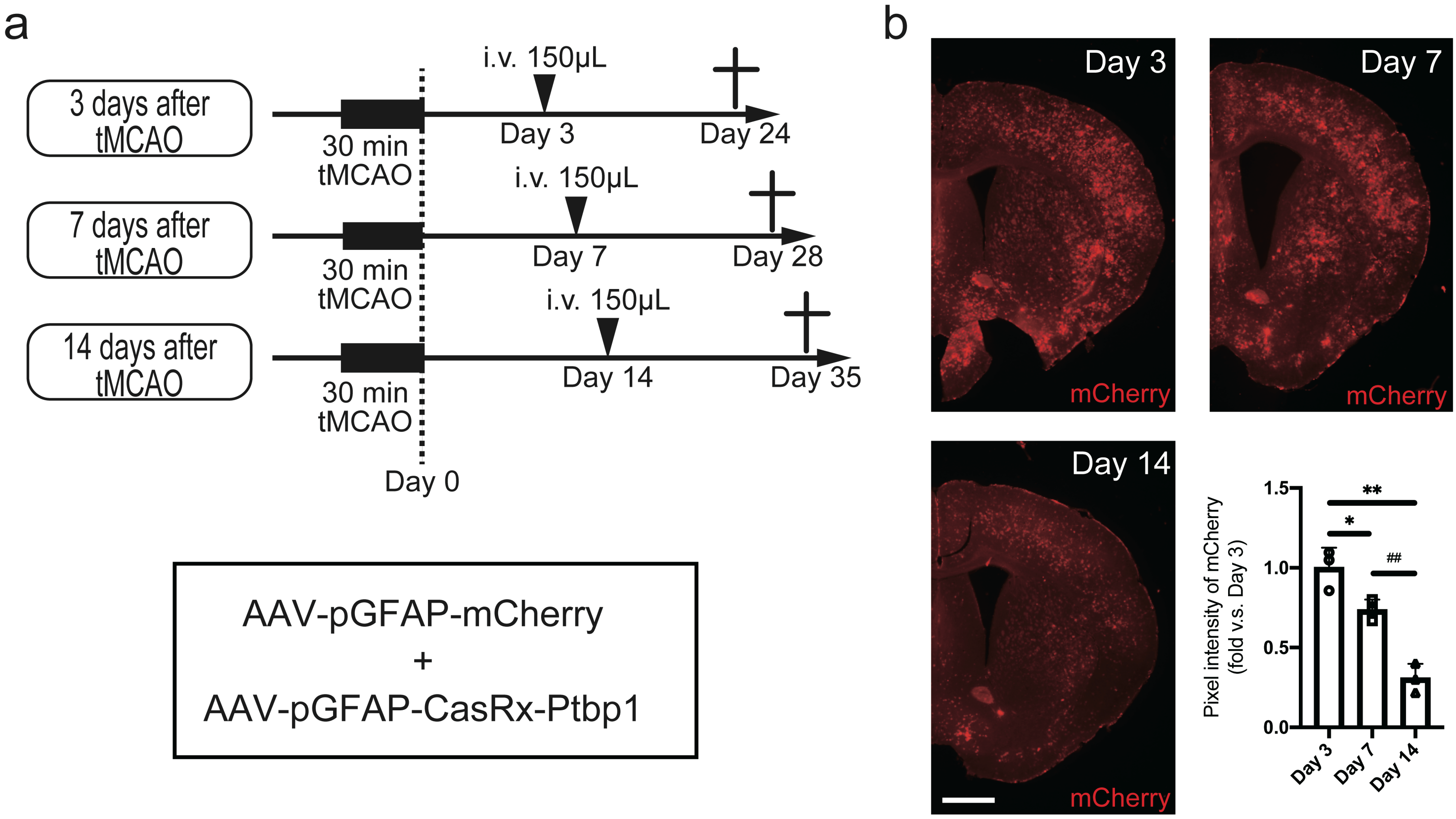

Fig. S2 (a) Schematic diagram of the experimental procedure to determine the optimal time point of the viral vector injection. The arrowheads indicate the intravenous injection of a cocktail of AAV (PHP.eB)-pGFAP-mCherry and AAV(PHP.eB)-pGFAP-CasRx-SgRNA-Ptbp1 (1:5, total 1.2×10^{10} vg) through the tail vein at 3, 7, or 14 days after 30 min tMCAO. (b) Immunofluorescent analysis of mCherry in the ipsilateral side of a stroke brain at 3, 7, or 14 days after tMCAO. The pixel intensity of mCherry showed a significant time-dependent decrease on day 7 and day 14. (*p<0.05). P-values < 0.05 were considered statistically significant. Scale bars: 1 mm.

Fig. S3

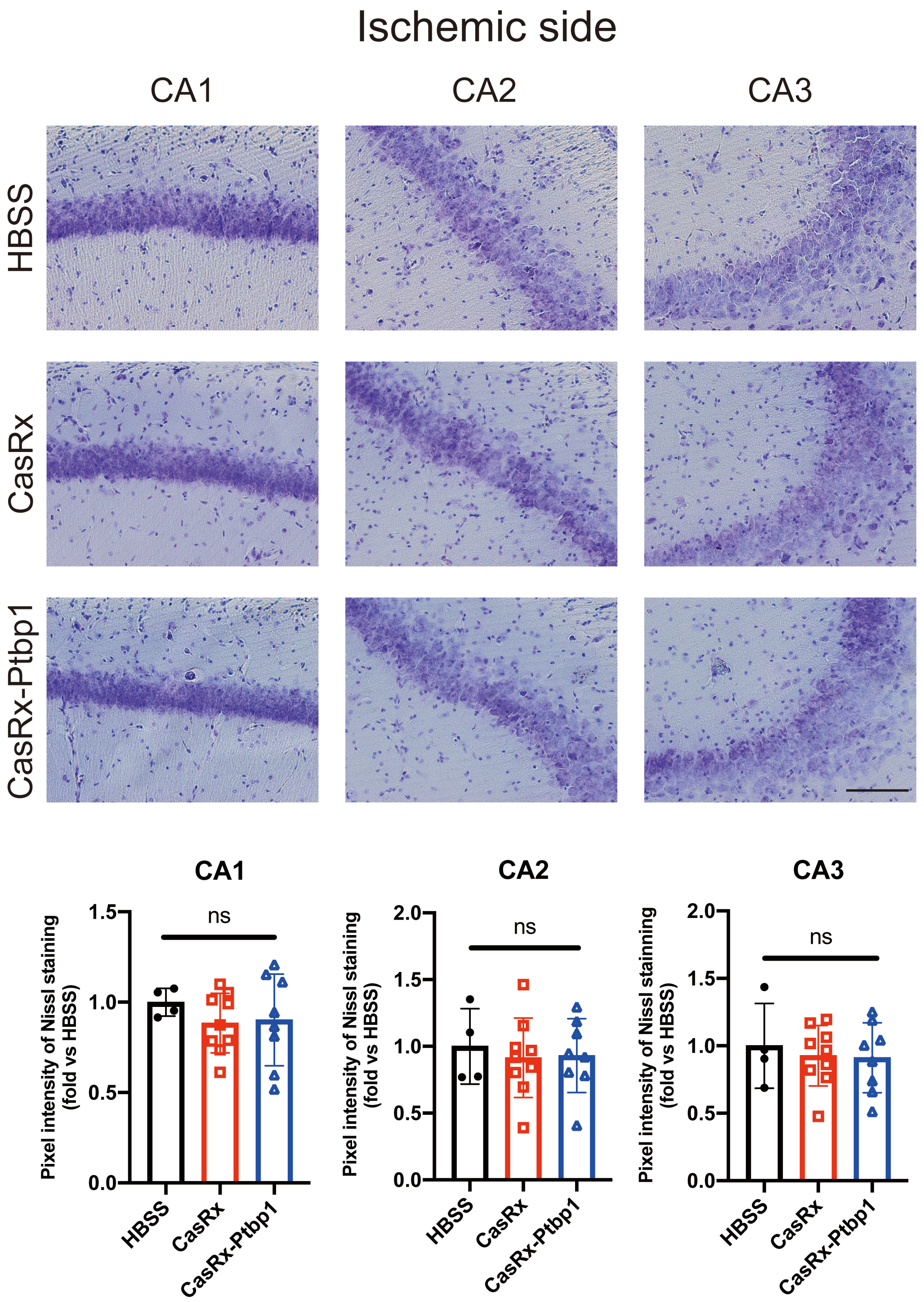

Fig. S3 Nissl staining was performed 56 days after tMCAO in the ischemic side of the hippocampal CA1, CA2, and CA3 regions, and the pixel intensity was calculated. There were no significant differences in Nissl staining in the ischemic side between the three groups. Scale bars: 100 μm.

Fig. S4

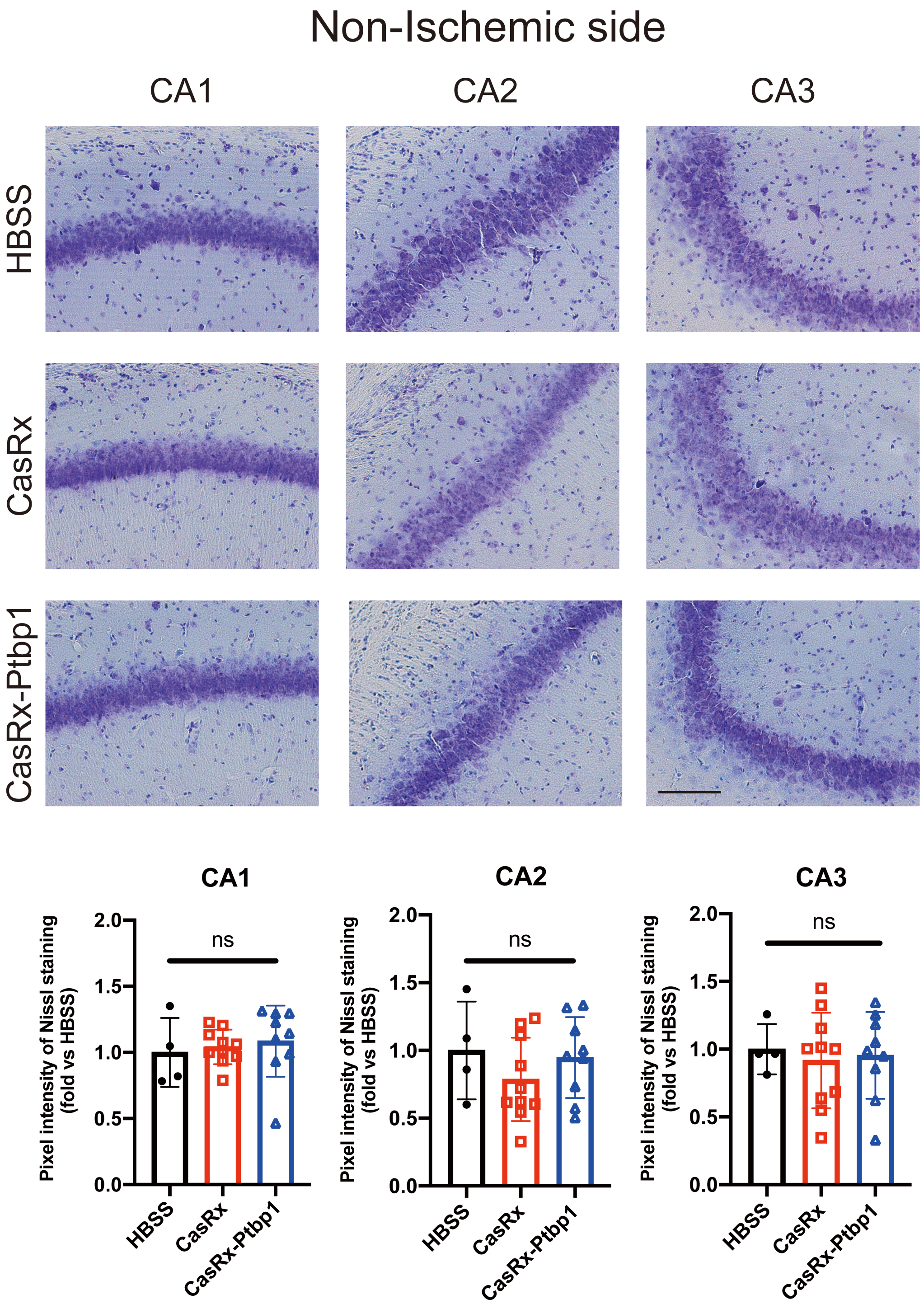

Fig. S4 Nissl staining was performed 56 days after tMCAO in the non-ischemic side of the hippocampal CA1, CA2, and CA3 regions, and the pixel intensity was calculated. There were no significant differences in Nissl staining in the non-ischemic side between the three groups. Scale bars: 100 μm.
